# Distinguishability and Disturbance in the Quantum Key Distribution Protocol Using the Mean Multi-Kings’ Problem

**DOI:** 10.3390/e22111275

**Published:** 2020-11-11

**Authors:** Masakazu Yoshida, Ayumu Nakayama, Jun Cheng

**Affiliations:** 1 Faculty of Design Technology, Osaka Sangyo University, 3-1-1 Daito-shi, Osaka 574-8530, Japan; 2 Independent Researcher, Chiba 263-8522, Japan; chiba.u.nakayama@gmail.com; 3 Faculty of Science and Engineering, Doshisha University, 1-3 Kyotanabe-shi, Kyoto 610-0394, Japan; jcheng@mail.doshisha.ac.jp

**Keywords:** quantum key distribution, mean-king’s problem, mean multi-kings’ problem, information disturbance theorem

## Abstract

We introduce a quantum key distribution protocol using mean multi-kings’ problem. Using this protocol, a sender can share a bit sequence as a secret key with receivers. We consider a relation between information gain by an eavesdropper and disturbance contained in legitimate users’ information. In BB84 protocol, such relation is known as the so-called information disturbance theorem. We focus on a setting that the sender and two receivers try to share bit sequences and the eavesdropper tries to extract information by interacting legitimate users’ systems and an ancilla system. We derive trade-off inequalities between distinguishability of quantum states corresponding to the bit sequence for the eavesdropper and error probability of the bit sequence shared with the legitimate users. Our inequalities show that eavesdropper’s extracting information regarding the secret keys inevitably induces disturbing the states and increasing the error probability.

## 1. Introduction

In the quantum state discrimination problems, one tries to discriminate the quantum states by performing the single measurement. Several strategies exist, e.g., in [1,2,3] and Section 9.1.4 in [4]. On the other hand, in the mean-king’s problem [5], one can use not the single measurement but also post-information. Specific setting of the mean-king’s problem is often told as a tale [6] of a king and a physicist Alice. In the tale, Alice prepares a qubit in an initial state at first. The king performs a measurement with one of observables $\sigma_{x},\sigma_{y},\sigma_{z}$ on the qubit and obtains an outcome. Then, Alice obtains an outcome by performing a measurement on the qubit. After the measurement, the king reveals the observable he has measured as the post-information. Then, Alice tries to guess king’s outcome by using her outcome and the post-information. A solution to the problem is a pair of the initial state and Alice’s measurement such that she can guess king’s outcome correctly. Using Aharonov–Bergman–Lebowitz rule [7], a solution which consists of Bell state and a measurement on a bipartite system has been shown [5]. As an application of the solution to the mean-king’s problem, a quantum key distribution protocol (QKD) has been shown [8]. In this protocol, Alice and the king employ the guessing result as a secret key, and security analysis of the protocol has been considered [8,9,10,11].

A QKD protocol by using mean multi-kings’ problem has been shown [12] (see Section 2 for details). In this protocol, Alice and kings (called King${}_{1}$, King${}_{2}$, …, King${}_{n}$) are legitimate users. Alice guesses each king’s measurement outcome by using her measurement outcome and post-information from each king; then, each guessing result is shared as a secret key between Alice and each king. The protocol has superior aspects, such as the number of measurements, state preparation and key discarding, to several realizations (whose components are the QKD protocol by using the mean-king’s problem or BB84 protocol [13]) for Alice and each king to share the secret key. In the case of n=2, security analysis against a simple attack so called intercept-resend attack has been considered and error rate of bits shared between Alice and the kings has been shown.

In this paper, we consider a relation between information gain by an eavesdropper (called Eve) and disturbance contained in the legitimate users’ information in the QKD protocol by using the mean multi-kings’ problem. In BB84 protocol, such relation is known as the so-called information disturbance theorem [14,15,16,17,18]. According to the theorem, Eve’s information gain in a basis inevitably induces disturbance contained in the legitimate users’ information in the conjugate basis. Therefore, the theorem is also regarded as an information theoretical version of the uncertainty relation. The theorem also plays an important role in the proof of the unconditional security [19]. We consider that Eve tries to extract information by employing an attack which she performs any measurement on her quantum system at any time after interacting the quantum system with kings’ qubits after their measurements in the case of n=2. In this setting, we give trade-off inequalities between distinguishability of quantum states corresponding to the bit sequences for Eve and error probability of the bit sequences shared with Alice and the kings. Our inequalities show that Eve’s extracting information regarding the secret keys inevitably induces disturbing the states of kings’ qubits and increasing the error probability even though the post-information and Alice’s qubit are used in the guessing step, unlike BB84 protocol.

This paper is organized as follows. In the next section, we review a description of the quantum key distribution protocol by using the mean multi-kings’ problem. In Section 3, we give the description of the protocol in the case of n=2. In Section 4, we give the outline of the attack and the trade-off inequalities between distinguishability and disturbance. Finally, we summarize this paper in Section 5.

## 2. Protocol

Let us start by introducing the essence of the mean multi-kings’ problem and the QKD by using it. Alice and King${}_{1}$, King${}_{2}$, …, King${}_{n}$ are the characters in this problem. The problem can be summarized as follows. Alice prepares a composite system, which consists of her system and *n* systems for kings, in an initial state. Each king performs a measurement on his system and obtains an outcome. After kings’ measurement, Alice performs a measurement on the composite system and obtains an outcome. Furthermore, each king reveals post-information: the measurement type he has performed. Immediately, Alice guesses kings’ outcomes by using her outcome and the post-information from each king. A solution to the problem is defined as a three-tuple of the initial state, Alice’s measurement, and a guessing function such that she can guess kings’ outcomes correctly. In this problem, the initial state will be changed depending on the kings’ measurements and outcomes. In general, it is impossible to distinguish the changed states correctly. Therefore, Alice tries to get some potential answers by performing the measurement and to narrow down the correct outcome from them by using the guessing function of her outcome and the post-information.

**Figure 1:**
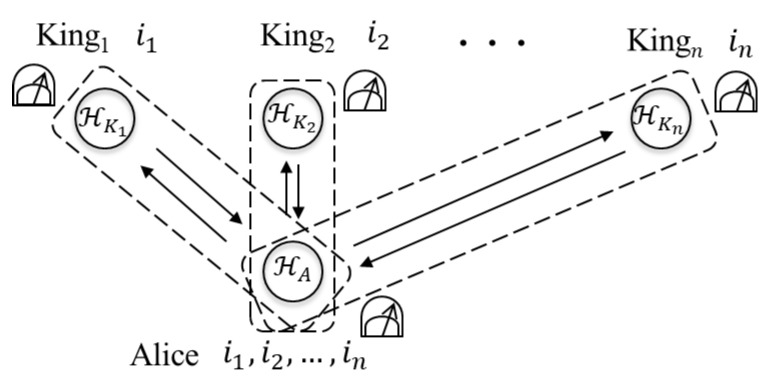
The QKD protocol by using the mean multi-kings’ problem.

We can construct the QKD protocol by using a setting of the mean multi-kings’ problem and a solution to it, i.e., Alice and each king share the guessing result as a secret key. Figure 1 is a graphically demonstrated protocol. Let us consider a setting that Alice prepares a composite system which consists of n+1 qubits and each king performs one of two fixed measurements on his qubit. Then, two solutions where the initial states are multipartite entangled states can be shown as described below; therefore, we can also construct the QKD protocol by using those solutions. In the QKD, Alice and each king try to share secret keys while she switches the solutions.

Before introducing details of the QKD protocol, we introduce some preliminary definitions, the setting of the mean multi-kings’ problem, and the solutions to it. Define

(1) $$Z_{0}:=|0\rangle \langle 0|,Z_{1}:=|1\rangle \langle 1|,X_{0}:=|\bar{0}\rangle \langle \bar{0}|,X_{1}:=|\bar{1}\rangle \langle \bar{1}|$$

for $|0\rangle :={\left(1,0\right)}^{T},|1\rangle :={\left(0,1\right)}^{T},|\bar{0}\rangle :=\frac{1}{\sqrt{2}}{\left(1,1\right)}^{T},|\bar{1}\rangle :=\frac{1}{\sqrt{2}}{\left(1,-1\right)}^{T}$. Define an outcome set

(2) $$K:=\{\left(s_{1},t_{1},s_{2},t_{2},\ldots ,s_{n},t_{n}\right)|s_{j},t_{j}\in \left\{0,1\right\}\},$$

operators for $(s_{1},t_{1},s_{2},t_{2},\ldots ,s_{n},t_{n})\in K$

(3) $$\begin{array}{ccc} E_{\left(s_{1},t_{1},s_{2},t_{2},\ldots ,s_{n},t_{n}\right)}^{\left(Z\right)} & := & X_{s_{1}}Z_{t_{1}}⊗X_{s_{2}}Z_{t_{2}}⊗\cdots ⊗X_{s_{n}}Z_{t_{n}}, \end{array}$$

(4) $$\begin{array}{ccc} E_{\left(s_{1},t_{1},s_{2},t_{2},\ldots ,s_{n},t_{n}\right)}^{\left(X\right)} & := & Z_{s_{1}}X_{t_{1}}⊗Z_{s_{2}}X_{t_{2}}⊗\cdots ⊗Z_{s_{n}}X_{t_{n}}, \end{array}$$

and an index set

(5) $$S_{{\left(J_{j},i_{j}\right)}_{j=1}^{n}}^{\left(W\right)}=S_{\left(J_{1},i_{1},J_{2},i_{2},\ldots ,J_{n},i_{n}\right)}^{\left(W\right)}:=S_{\left(J_{1},i_{1}\right)}^{\left(W\right)}\times S_{\left(J_{2},i_{2}\right)}^{\left(W\right)}\times \cdots \times S_{\left(J_{n},i_{n}\right)}^{\left(W\right)}$$

(W∈{Z,X}) which consists of direct product of

(6) $$\begin{array}{c} S_{\left(J,i\right)}^{\left(Z\right)}:=\left\{\begin{array}{c} \left\{(0,i),(1,i)\}\;(J=0,i\in \{0,1\}\right)\\ \{(i,0),(i,1)\}\;(J=1,i\in \{0,1\}), \end{array}\right. \end{array}$$

(7) $$\begin{array}{c} S_{\left(J,i\right)}^{\left(X\right)}:=\left\{\begin{array}{c} \left\{(i,0),(i,1)\}\;(J=0,i\in \{0,1\}\right)\\ \{(0,i),(1,i)\}\;(J=1,i\in \{0,1\}). \end{array}\right. \end{array}$$

We define the setting of the mean multi-kings’ problem. Alice prepares the composite system (n+1 qubits) $\tilde{H}:=H_{A}⊗H_{K_{1}}⊗H_{K_{2}}⊗\cdots ⊗H_{K_{n}}≃{\left(C^{2}\right)}^{⊗n+1}$ in an initial state. Each King${}_{j}$ performs one of the measurements on $H_{K_{j}}$

(8) $$\begin{array}{c} M^{\left(J_{j}\right)}=\left(M_{0}^{\left(J_{j}\right)},M_{1}^{\left(J_{j}\right)}\right)\;\;\left(J_{j}\in \left\{0,1\right\}\right), \end{array}$$

where $M^{\left(0\right)}:=\left(M_{0}^{\left(0\right)}:=Z_{0},M_{1}^{\left(0\right)}:=Z_{1}\right)$ and $M^{\left(1\right)}:=\left(M_{0}^{\left(1\right)}:=X_{0},M_{1}^{\left(1\right)}:=X_{1}\right)$, and obtains an outcome $i_{j}\in \left\{0,1\right\}$. Alice performs a measurement on $\tilde{H}$ and obtains an outcome. After Alice’s measurement, the kings reveal ${\left(J_{j}\right)}_{j=1}^{n}$ as the post-information. Then, Alice tries to guess kings’ outcomes by using her outcome and the post-information.

Here, we show two solutions to the problem. In this case, Alice can guess the kings’ outcomes correctly by employing one of

(9) $$\begin{array}{c} |\Phi^{\left(Z\right)}\rangle :=\frac{1}{\sqrt{2}}(|00\cdots 0\rangle +\left|11\cdots 1\rangle \right) \end{array}$$

(10) $$\begin{array}{c} |\Phi^{\left(X\right)}\rangle :=\frac{1}{\sqrt{2}}(|\bar{0}\bar{0}\cdots \bar{0}\rangle +|\bar{1}\bar{1}\cdots \bar{1}\rangle ) \end{array}$$

as an initial state, a measurement depending on the initial state $|\Phi^{\left(W\right)}\rangle$

(11) $$P^{\left(W\right)}:={\left(P_{k}^{\left(W\right)}:=2^{n+1}|\left(I⊗E_{k}^{\left(W\right)}\right)\Phi^{\left(W\right)}\rangle \langle \left(I⊗E_{k}^{\left(W\right)}\right)\Phi^{\left(W\right)}|\right)}_{k\in K}$$

and a guessing function $s(k,{\left(J_{j}\right)}_{j=1}^{n},\Phi^{\left(W\right)})$ of her outcome k∈K, the post-information ${\left(J_{j}\right)}_{j=1}^{n}$, and the initial state $|\Phi^{\left(W\right)}\rangle$, where $s(k,{\left(J_{j}\right)}_{j=1}^{n},\Phi^{\left(W\right)})$ is defined as ${\left(i_{j}\right)}_{j=1}^{n}$ satisfying $k\in S_{{\left(J_{j},i_{j}\right)}_{j=1}^{n}}^{\left(W\right)}$ (we regard $k=(s_{1},t_{1},s_{2},t_{2},\ldots ,s_{n},t_{n})$ in the same light as $\left(\left(s_{1},t_{1}\right),\left(s_{2},t_{2}\right),\ldots ,\left(s_{n},t_{n}\right)\right)$).

We clear the number of non-zero matrices in her measurement and their orthogonality. We can observe

(12) $$\begin{array}{ccc} |\left(I⊗E_{k}^{\left(Z\right)}\right)\Phi^{\left(Z\right)}\rangle \; & = & \left(I⊗X_{s_{1}}Z_{t_{1}}⊗\cdots ⊗X_{s_{n}}Z_{t_{n}}\right)\frac{1}{\sqrt{2}}(|00\cdots 0\rangle +\left|11\cdots 1\rangle \right)\\ \; & = & \frac{1}{\sqrt{2}}(\delta_{t_{1}0}\cdots \delta_{t_{n}0}|0\rangle X_{s_{1}}|0\rangle ⊗X_{s_{2}}|0\rangle ⊗\cdots ⊗X_{s_{n}}|0\rangle \\ \; & \; & +\delta_{t_{1}1}\cdots \delta_{t_{n}1}|1\rangle ⊗X_{s_{1}}|1\rangle ⊗X_{s_{2}}|1\rangle ⊗\cdots ⊗X_{s_{n}}|1\rangle ). \end{array}$$

Then, the number of non-zero vectors is equal to $2^{n+1}$. It leads to the conclusion that the number of non-zero matrices in $P^{\left(Z\right)}$ is equal to $2^{n+1}$. Furthermore, we observe

(13) $$\begin{array}{ccc} \; & \; & \langle \left(I⊗E_{k}^{\left(Z\right)}\right)\Phi^{\left(Z\right)}|\left(I⊗E_{k^{\prime }}^{\left(Z\right)}\right)\Phi^{\left(Z\right)}\rangle \\ \; & = & \langle \left(I⊗X_{s_{1}}Z_{t_{1}}⊗\cdots ⊗X_{s_{n}}Z_{t_{n}}\right)\Phi^{\left(Z\right)}|\left(I⊗X_{s_{1}^{\prime }}Z_{t_{1}^{\prime }}⊗\cdots ⊗X_{s_{n}^{\prime }}Z_{t_{n}^{\prime }}\right)\Phi^{\left(Z\right)}\rangle \\ \; & = & \frac{1}{2^{n+1}}\left(\delta_{t_{1}0}\delta_{t_{2}0}\cdots \delta_{t_{n}0}+\delta_{t_{1}1}\delta_{t_{2}1}\cdots \delta_{t_{n}1}\right)\delta_{kk^{\prime }}. \end{array}$$

It implies that $P^{\left(Z\right)}$ is an orthogonal measurement on $\tilde{H}$. When *Z* is switched to *X*, we have the same result in the case of W=X.

Next, we show that Alice can correctly guess kings’ outcomes. We observe

(14) $$\begin{array}{c} S_{{\left(J_{j},i_{j}\right)}_{j=1}^{n}}^{\left(W\right)}\cap S_{{\left(J_{j},i_{j}^{\prime }\right)}_{j=1}^{n}}^{\left(W\right)}=\emptyset \end{array}$$

for any $J_{j}$ and $\left(i_{1},i_{2},\ldots ,i_{n}\right)\neq \left(i_{1}^{\prime },i_{2}^{\prime },\ldots ,i_{n}^{\prime }\right)$, and

(15) $$\begin{array}{c} M_{i_{1}}^{\left(J_{1}\right)}⊗M_{i_{2}}^{\left(J_{2}\right)}⊗\cdots ⊗M_{i_{n}}^{\left(J_{n}\right)}=\sum_{k\in S_{{\left(J_{j},i_{j}\right)}_{j=1}^{n}}^{\left(W\right)}}E_{k}^{\left(W\right)} \end{array}$$

holds for any $J_{j}$ and $i_{j}$. When King${}_{j}$ performs the measurement $M^{\left(J_{j}\right)}$ and obtains an outcome $i_{j}$, by Equation (15), the post-measurement state is proportional to

(16) $$\begin{array}{c} |\left(I⊗M_{i_{1}}^{\left(J_{1}\right)}⊗M_{i_{2}}^{\left(J_{2}\right)}⊗\cdots ⊗M_{i_{n}}^{\left(J_{n}\right)}\right)\Phi^{\left(W\right)}\rangle \in \underset{k\in S_{{\left(J_{j},i_{j}\right)}_{j=1}^{n}}^{\left(W\right)}}{⨁}A_{k}, \end{array}$$

where $A_{k}$ is a subspace spanned by $|\left(I⊗E_{k}^{\left(W\right)}\right)\Phi^{\left(W\right)}\rangle$. $A_{k}$ and $A_{k^{\prime }}$ are orthogonal for any $k\neq k^{\prime }$ and $P^{\left(W\right)}$ is composed of orthogonal projections onto each subspace $A_{k}$ by Equation (13). If Alice obtains an outcome *k* by performing $P^{\left(W\right)}$ and the post-information ${\left(J_{j}\right)}_{j=1}^{n}$ from the kings, then kings’ outcomes ${\left(i_{j}\right)}_{j=1}^{n}$ should satisfy $k\in S_{{\left(J_{j},i_{j}\right)}_{j=1}^{n}}^{\left(W\right)}$. However, by Equation (14), such ${\left(i_{j}\right)}_{j=1}^{n}$ uniquely exists. Thus, Alice can correctly guess kings’ outcomes.

A description of the QKD protocol by using the mean multi-kings’ problem is as follows.

1. Alice prepares a composite system (n+1 qubits) $\tilde{H}=H_{A}⊗H_{K_{1}}⊗H_{K_{2}}⊗\cdots ⊗H_{K_{n}}≃{\left(C^{2}\right)}^{⊗n+1}$ in the initial state $|\Phi^{\left(W\right)}\rangle$ (W∈{Z,X}) with probability $\frac{1}{2}$. Then, she sends the qubit $H_{K_{j}}$ to King${}_{j}$ (j=1,2,…,n).
2. Each King${}_{j}$ performs the measurement $M^{\left(J_{j}\right)}=\left(M_{0}^{\left(J_{j}\right)},M_{1}^{\left(J_{j}\right)}\right)$ ($J_{j}\in \left\{0,1\right\}$) with probability $\frac{1}{2}$ on $H_{K_{j}}$ and obtains an outcome $i_{j}\in \left\{0,1\right\}$. After the measurement, each King${}_{j}$ returns $H_{K_{j}}$ to Alice.
3. Alice performs the measurement $P^{\left(W\right)}={\left(P_{k}^{\left(W\right)}\right)}_{k\in K}$ (W∈{Z,X}) on $\tilde{H}$ when the initial state was $|\Phi^{\left(W\right)}\rangle$. Then, she obtains an outcome k∈K.
4. After the measurement, each King${}_{j}$ announces post-information $J_{j}$ to Alice.
5. Alice obtains a sequence $s(k,{\left(J_{j}\right)}_{j=1}^{n},\Phi^{\left(W\right)})$ from the outcome *k*, the post-information ${\left(J_{j}\right)}_{j=1}^{n}$, and the initial state $|\Phi^{\left(W\right)}\rangle$.
6. They repeat the above process. After that, Alice randomly chooses sequences ${\left(i_{j}^{\prime 1}\right)}_{j=1}^{n},{\left(i_{j}^{\prime 2}\right)}_{j=1}^{n},\ldots ,{\left(i_{j}^{\prime r}\right)}_{j=1}^{n}$ from all sequences. Similarly, kings work together to choose sequences ${\left(i_{j}^{1}\right)}_{j=1}^{n},{\left(i_{j}^{2}\right)}_{j=1}^{n},\ldots ,{\left(i_{j}^{r}\right)}_{j=1}^{n}$ which are the same positions as the positions Alice chose. Then, Alice and kings work together to calculate error rate $\frac{\sum_{u=1}^{r}\left(1-\delta_{{\left(i_{j}^{\prime u}\right)}_{j=1}^{n}{\left(i_{j}^{u}\right)}_{j=1}^{n}}\right)}{r}$.

The rest of the process is the same as for ordinary QKD protocols, such as BB84 protocol. If the error rate is too large, the protocol is aborted. Otherwise, the leftover sequences are performed with error-correction and privacy amplification [20].

**Figure 2:**
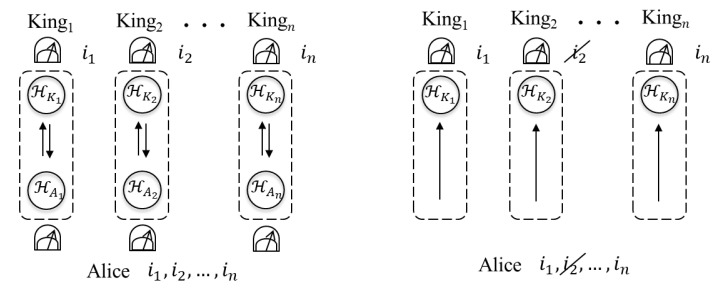
The QKD protocols using the mean-king’s problem (**left hand side**) and BB84 protocols (**right hand side**) for Alice and the kings to share the secret key.

Remark that Alice and each King${}_{j}$ can share the secret key when they employ the QKD protocol using the mean-king’s problem or BB84 protocol. In the case of employing the QKD using the mean-king’s problem (see left hand side of Figure 2), Alice prepares 2 qubits in the Bell state and performs a single measurement on the 2 qubits for each King${}_{j}$. Therefore, she needs to prepare 2n qubits and perform *n* measurements to share the secret key with *n* kings. On the other hand, in the QKD protocol using the mean multi-kings’ problem, Alice only prepares n+1 qubits in $|\Phi^{\left(Z\right)}\rangle$ or $|\Phi^{\left(X\right)}\rangle$ and performs the single measurement $P^{\left(Z\right)}$ or $P^{\left(X\right)}$. In the case where the BB84 protocol is employed (see right hand side of Figure 2), Alice just prepares *n* qubits in one of the states $\left|0\rangle ,|1\rangle ,\right|\bar{0}\rangle ,|\bar{1}\rangle$ and no performing the measurement is required. Then, Alice and King${}_{j}$ discard the raw key where their bases do not match before calculating error rate. On the other hand, in the QKD protocol using the mean multi-kings’ problem, there is not such discarding step before calculating error rate.

## 3. Protocol: *n* = 2

We describe the working of the protocol in the case of n=2 by focusing on the case of W=Z to reduce cumbersome notations.

By Equation (2), the index set takes the following form,

(17) $$K=\{\left(s_{1},t_{1},s_{2},t_{2}\right)|s_{j},t_{j}\in \left\{0,1\right\}\}.$$

And by Equation (3), the operator $E_{k}^{\left(Z\right)}$ for k∈K takes the following form,

(18) $$E_{k}^{\left(Z\right)}=E_{\left(s_{1},t_{1},s_{2},t_{2}\right)}^{\left(Z\right)}=X_{s_{1}}Z_{t_{1}}⊗X_{s_{2}}Z_{t_{2}}\;\;\left(k=\left(s_{1},t_{1},s_{2},t_{2}\right)\in K\right).$$

Similarly, we can observe the operators for W=X. By Equation (5), we observe the index sets $S_{\left(J_{1},i_{1},J_{2},i_{2}\right)}^{\left(W\right)}$ for $J_{1}=0,J_{2}=0,i_{1},i_{2}\in \left\{0,1\right\}$, and W=Z:

(19) $$\begin{array}{ccc} S_{\left(0,0,0,0\right)}^{\left(Z\right)} & = & S_{\left(0,0\right)}^{\left(Z\right)}\times S_{\left(0,0\right)}^{\left(Z\right)}\\ & = & \left\{((0,0),(0,0)),((0,0),(1,0)),((1,0),(0,0)),((1,0),(1,0))\right\}\\ & = & \left\{(0,0,0,0),(0,0,1,0),(1,0,0,0),(1,0,1,0)\right\} \end{array}$$

(20) $$\begin{array}{ccc} S_{\left(0,0,0,1\right)}^{\left(Z\right)} & = & S_{\left(0,0\right)}^{\left(Z\right)}\times S_{\left(0,1\right)}^{\left(Z\right)}\\ & = & \left\{((0,0),(0,1)),((0,0),(1,1)),((1,0),(0,1)),((1,0),(1,1))\right\}\\ & = & \left\{(0,0,0,1),(0,0,1,1),(1,0,0,1),(1,0,1,1)\right\} \end{array}$$

(21) $$\begin{array}{ccc} S_{\left(0,1,0,0\right)}^{\left(Z\right)} & = & S_{\left(0,1\right)}^{\left(Z\right)}\times S_{\left(0,0\right)}^{\left(Z\right)}\\ & = & \left\{((0,1),(0,0)),((0,1),(1,0)),((1,1),(0,0)),((1,1),(1,0))\right\}\\ & = & \left\{(0,1,0,0),(0,1,1,0),(1,1,0,0),(1,1,1,0)\right\} \end{array}$$

(22) $$\begin{array}{ccc} S_{\left(0,1,0,1\right)}^{\left(Z\right)} & = & S_{\left(0,1\right)}^{\left(Z\right)}\times S_{\left(0,1\right)}^{\left(Z\right)}\\ & = & \left\{((0,1),(0,1)),((0,1),(1,1)),((1,1),(0,1)),((1,1),(1,1))\right\}\\ & = & \{(0,1,0,1),(0,1,1,0),(1,1,0,1),(1,1,1,1)\}, \end{array}$$

where we regard $\left(\left(l_{1},l_{2}\right),\left(l_{3},l_{4}\right)\right)$ in the same light as $\left(l_{1},l_{2},l_{3},l_{4}\right)$. Similarly, we can observe the index sets for other $J_{1},J_{2},i_{1},i_{2}$, and *W*.

Let us consider that Alice prepares the qubits $\tilde{H}=H_{A}⊗H_{K_{1}}⊗H_{K_{2}}$ in the initial state

(23) $$|\Phi^{\left(Z\right)}\rangle =\frac{1}{\sqrt{2}}(|000\rangle +|111\rangle ).$$

Let us consider that King${}_{1}$ and King${}_{2}$ choose the same measurement $M^{\left(0\right)}$ and obtain the same outcome 0, i.e., $J_{1}=0,J_{2}=0$ and $i_{1}=0,i_{2}=0$. After kings’ measurement, Alice performs the measurement $P^{\left(Z\right)}={\left(P_{k}^{\left(Z\right)}\right)}_{k\in K}$ on $\tilde{H}$, where

(24) $$\begin{array}{ccc} P_{k}^{\left(Z\right)} & = & 8|\left(I⊗E_{k}^{\left(Z\right)}\right)\Phi^{\left(Z\right)}\rangle \langle \left(I⊗E_{k}^{\left(Z\right)}\right)\Phi^{\left(Z\right)}|r\\ \; & = & 8|\left(I⊗X_{s_{1}}Z_{t_{1}}⊗X_{s_{2}}Z_{t_{2}}\right)\Phi^{\left(Z\right)}\rangle \langle \left(I⊗X_{s_{1}}Z_{t_{1}}⊗X_{s_{2}}Z_{t_{2}}\right)\Phi^{\left(Z\right)}|. \end{array}$$

After the measurement, King${}_{1}$ and King${}_{2}$ announce the post-information $J_{1}=0$ and $J_{2}=0$ to Alice. When Alice obtains an outcome k=(0,0,0,0), she is assured that kings’ outcome $\left(i_{1},i_{2}\right)$ is (0,0), because $\left(i_{1},i_{2}\right)$ satisfying $k=\left(0,0,0,0\right)\in S_{\left(J_{1},i_{1},J_{2},i_{2}\right)}^{\left(W\right)}=S_{\left(0,i_{1},0,i_{2}\right)}^{\left(Z\right)}$ is (0,0). In Table 1, we summarize Alice’s guessing rule by using her outcome and the post-information from the kings.

In the case of n=2, the following simple attack so called intercept-resend attack can be considered. An eavesdropper (called Eve) intercepts $H_{K_{j}}$ returned to Alice from King${}_{j}$ (step 2 in the protocol) and performs the measurement $M^{\left(0\right)}$ or $M^{\left(1\right)}$ probabilistically on $H_{K_{j}}$. After the measurement, she resends $H_{K_{j}}$ to Alice. When Eve performs the intercept-resend attack to only $H_{K_{1}}$, the probability which the error occurs is $\frac{1}{8}$, where the error means the event: $\delta_{{\left(i_{j}^{\prime u}\right)}_{j=1}^{2}{\left(i_{j}^{u}\right)}_{j=1}^{2}}=0$. When Eve performs the intercept-resend attack to both $H_{K_{1}}$ and $H_{K_{2}}$, the probability which the error occurs is $\frac{1}{32}\left(p_{1}+p_{2}-2p_{1}p_{2}+7\right)$, where $p_{j}$ denotes the probability, which Eve performs the measurement $M^{\left(0\right)}$ on $H_{K_{j}}$ (j∈{1,2}). The minimum value of the probability is 0.21875 when $\left(p_{1}=1,p_{2}=1\right)$ or $\left(p_{1}=0,p_{2}=0\right)$ and the maximum value of the probability is 0.25 when $\left(p_{1}=1,p_{2}=0\right)$ or $\left(p_{1}=0,p_{2}=1\right)$.

## 4. Distinguishability vs. Disturbance

**Figure 3:**
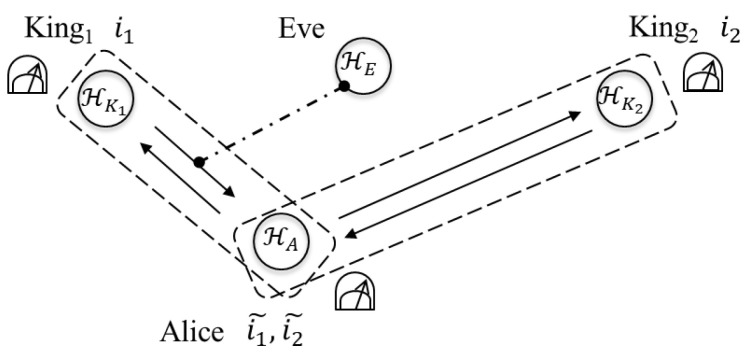
The interaction $H_{K_{1}}$ with $H_{E}$.

**Figure 4:**
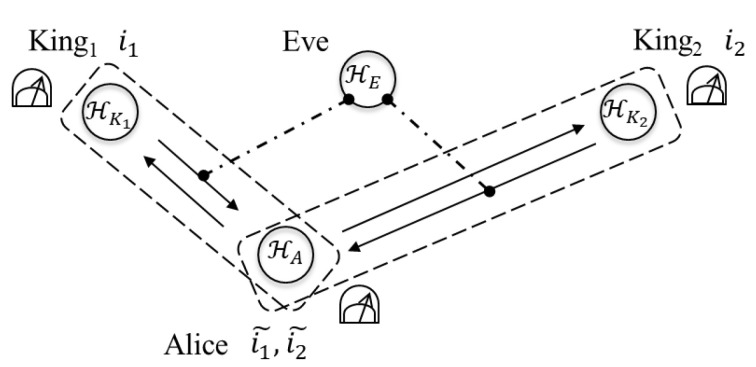
The interaction $H_{K_{1}}⊗H_{K_{2}}$ with $H_{E}$.

In this section, let us consider two types of the attacks and let us see whether Eve can extract information by employing the attacks without disturbing contained in legitimate users’ information in the case of n=2. First, Eve tries to gain information from the qubit returned to Alice by King${}_{1}$ (step 2 in the protocol) by interacting the qubit $H_{K_{1}}$ with her quantum system $H_{E}$ (see Figure 3). Second, she tries to gain information from the qubits $H_{K_{j}}$ returned to Alice by King${}_{j}$ (step 2 in the protocol) by interacting $H_{K_{1}}⊗H_{K_{2}}$ with her quantum system $H_{E}$ (see Figure 4). In both of the attacks, Eve performs any measurement on her quantum system $H_{E}$ at any time.

We can consider an attack that Eve interacts her quantum system with the qubits sent to the kings by Alice. However, in this attack, the qubits are not encoded because the kings have not measured the qubits. Especially, in the case of n=1, the setting of the attack can be considered as monogamy of entanglement [21,22]. Moreover, we can also consider an attack that Eve interacts her quantum system with both of the qubits sent to the kings by Alice and the qubits returned to Alice by the kings. However, the setting of the attack is different from one for discussing the information disturbance theorem. In the setting for the theorem, Eve tries to information extract from only the encoded qubits. Therefore, we concentrate on the above two attacks that Eve tries to extract information from the qubits sent to Alice by the kings.

In the beginning, we define error probability which represents probability that Alice cannot guess king’s outcomes correctly by using her outcome and the post-information. Remark that the error probability is different from the error rate (step 6 in the protocol). Let $P^{\left(W\right)}\left(k|J_{1};i_{1},J_{2};i_{2}\right)$ be the probability that Alice obtains an outcome *k* when she chooses $|\Phi^{\left(W\right)}\rangle$ and King${}_{j}$ obtains an outcome $i_{j}$ with the measurement $M^{\left(J_{j}\right)}$ (j∈{1,2}). We define

(25) $$P_{\mathrm{suc}(J_{1};i_{1},J_{2};i_{2})}^{\left(W\right)}:=\sum_{k\in S_{{\left(J_{j},i_{j}\right)}_{j=1}^{2}}^{\left(W\right)}}P^{\left(W\right)}\left(k|J_{1};i_{1},J_{2};i_{2}\right)$$

and

(26) $$P_{\mathrm{suc}(J_{1};i_{1},J_{2};i_{2})}:=\frac{1}{2}\sum_{W\in \{X,Z\}}P_{\mathrm{suc}(J_{1};i_{1},J_{2};i_{2})}^{\left(W\right)}.$$

Then, we define the error probability when King${}_{j}$ obtains an outcome $i_{j}$ with the measurement $M^{\left(J_{j}\right)}$:

(27) $$P_{\mathrm{err}(J_{1};i_{1},J_{2};i_{2})}:=1-P_{\mathrm{suc}(J_{1};i_{1},J_{2};i_{2})}.$$

Equation (27) represents probability that Alice’s sequence and kings’ sequence do not match when King${}_{j}$ obtains an outcome $i_{j}$ with the measurement $M^{\left(J_{j}\right)}$, i.e., Alice cannot guess kings’ outcomes correctly by using her outcome and the post-information.

Let us consider that Eve tries to extract information from $H_{K_{1}}$. Eve prepares her own quantum system $H_{E}$ in a quantum state Ω. She intercepts $H_{K_{1}}$ in the state $\rho^{\left(K_{1}\right)}$ returned to Alice by King${}_{1}$ and interacts it with $H_{E}$. Let us denote the interaction by

(28) $$T^{*}\left(\rho^{\left(K_{1}\right)}\right):=U\rho^{\left(K_{1}\right)}⊗\Omega U^{†},$$

where *U* is a unitary operator on $H_{K_{1}}⊗H_{E}$. Moreover, we denote the local state of $H_{E}$ (resp. $H_{K_{1}}$) by partial trace over the $H_{K_{1}}$ (resp. $H_{E}$)

(29) $$T_{E}^{*}\left(\rho^{\left(K_{1}\right)}\right):={\mathrm{tr}}_{H_{K_{1}}}T^{*}\left(\rho^{\left(K_{1}\right)}\right)\;\;\left(\mathrm{resp}.\;T_{K_{1}}^{*}\left(\rho^{\left(K_{1}\right)}\right):={\mathrm{tr}}_{H_{K_{E}}}T^{*}\left(\rho^{\left(K_{1}\right)}\right)\right).$$

Let us consider that King${}_{1}$ obtains an outcome *i* with a measurement $M^{\left(1\right)}$. Then, the state of $H_{K_{1}}$ before the interaction is $\rho^{\left(K_{1}\right)}=|\bar{i}\rangle \langle \bar{i}|$. Eve tries to extract information regarding to the secret key by distinguishing $T_{E}^{*}(|\bar{0}\rangle \langle \bar{0}|)$ and $T_{E}^{*}(|\bar{1}\rangle \langle \bar{1}|)$.

We employ trace distance as a measure for distinguishability of the states. Trace norm between a state ρ and a state σ is defined as $||\rho -{\sigma ||}_{1}:=\sup_{||A||=1}\left|\mathrm{tr}\left(\rho -\sigma \right)A\right|$, where ||·|| denotes operator norm. Trace distance is defined as follows,

(30) $$D\left(\rho ,\sigma \right):=\frac{1}{2}||\rho -{\sigma ||}_{1}.$$

It takes a value from 0 to 1. In addition, D(ρ,σ)=0 if and only if ρ=σ, and D(ρ,σ)=0 if and only if tr(ρσ)=0. Let us remind the definition of fidelity [23,24]. Fidelity between ρ and σ is defined as $F\left(\rho ,\sigma \right):=\mathrm{tr}\sqrt{\rho^{1/2}\sigma \rho^{1/2}}$. The following alternative expression of fidelity [25,26] has been shown,

(31) $$\begin{array}{c} F\left(\rho ,\sigma \right)=\underset{{\left(M_{a}\right)}_{a}:\mathrm{POVM}}{\inf}\sum_{a}\sqrt{p(a|\rho )p(a|\sigma )}, \end{array}$$

where p(a∣ρ) and p(a∣σ) are defined as $p\left(a|\rho \right):=\mathrm{tr}\left(M_{a}\rho \right)$ and $p\left(a|\sigma \right):=\mathrm{tr}\left(M_{a}\sigma \right)$.

**Lemma** **1.** *The following relation between trace distance and fidelity holds,*

(32) $$\begin{array}{c} \frac{1}{2}\left|\right|T_{E}^{*}(|\bar{0}\rangle \langle \bar{0}|)-T_{E}^{*}(|\bar{1}\rangle \langle \bar{1}|){\left|\right|}_{1}\leq F\left(T_{K_{1}}^{*}(|0\rangle \langle 0|),T_{K_{1}}^{*}(|1\rangle \langle 1|)\right). \end{array}$$

**Proof of Lemma** **1.** From Lemma 3 in [27], we have

(33) $$\left|\langle 0|T\left(I⊗A\right)|1\rangle \right|\leq ||A||F\left(T_{K_{1}}^{*}(|0\rangle \langle 0|),T_{K_{1}}^{*}(|1\rangle \langle 1|)\right)$$

for any operator *A* on $H_{E}$, where *T* is defined as $\mathrm{tr}T^{*}\left(\rho \right)X=\mathrm{tr}\rho T\left(X\right)$. By using Equation (33), we observe

(34) $$\begin{array}{ccc} |\mathrm{tr}\left[\left\{T_{E}^{*}(|\bar{0}\rangle \langle \bar{0}|-T_{E}^{*}\left(\frac{1}{2}I\right)\right\}A\right]|\; & = & \left|\mathrm{tr}\left\{\left(|\bar{0}\rangle \langle \bar{0}|-\frac{1}{2}I\right)T\left(I⊗A\right)\right\}\right|\\ \; & = & \left|\mathrm{tr}\left\{\frac{1}{2}(|0\rangle \langle 1|+|1\rangle \langle 0|)T\left(I⊗A\right)\right\}\right|\\ \; & \leq & \frac{1}{2}\left\{|\langle 1|T\left(I⊗A\right)|0\rangle \right|+\left|\langle 0|T\left(I⊗A\right)|1\rangle |\right\}\\ \; & \leq & ||A||F(T_{K_{1}}^{*}(|0\rangle \langle 0|),T_{K_{1}}^{*}(|1\rangle \langle 1|)). \end{array}$$

Then,

(35) $$\begin{array}{ccc} \frac{1}{2}\left|\right|T_{E}^{*}(|\bar{0}\rangle \langle \bar{0}|)-T_{E}^{*}(|\bar{1}\rangle \langle \bar{1}|){\left|\right|}_{1}\; & = & ||T_{E}^{*}(|\bar{0}\rangle \langle \bar{0}|)-T_{E}^{*}\left(\frac{1}{2}I\right)|{|}_{1}\\ \; & = & \underset{||A||=1}{\sup}|\mathrm{tr}\left[\left\{(T_{E}^{*}(|\bar{0}\rangle \langle \bar{0}|)-T_{E}^{*}\left(\frac{1}{2}I\right)\right\}A\right]|\\ \; & \leq & F(T_{K_{1}}^{*}(|0\rangle \langle 0|),T_{K_{1}}^{*}(|1\rangle \langle 1|)) \end{array}$$

holds. □

**Theorem** **1.** *The following trade-off inequality holds,*

(36) $$\begin{array}{ccc} D(T_{E}^{*}(|\bar{0}\rangle \langle \bar{0}|),T_{E}^{*}(|\bar{1}\rangle \langle \bar{1}|)) & \leq & \sqrt{2P_{\mathrm{err}(0;0,0;0)}}+\sqrt{2P_{\mathrm{err}(0;1,0;1)}}. \end{array}$$

The left hand side of the inequality represents distinguishability for Eve, and the right hand side is the sum of the error probabilities which represent probability that Alice’s sequence and kings’ sequence are not equal when the kings obtain the corresponding outcomes with the corresponding measurements, i.e., Alice cannot guess kings’ sequence correctly by using her outcome and the post-information. This theorem shows that Eve’s extracting information regarding King${}_{1}$’s key related with the measurement $M^{\left(1\right)}$ inevitably induces disturbing the states and increases the error probability when both of kings choose the measurement $M^{\left(0\right)}$. This implies that the more Eve extracts information, the more possibility for Alice and the kings to detect the existence of the attack increases. In particular, Eve cannot extract information about the key at all (i.e., trace distance is zero) when the corresponding error probabilities are zero. Remark that similar inequalities between distinguishability of other pairs of states and the error probabilities can be proven in the similar way as below.

**Proof of Theorem** **1.** Before obtaining the inequalities, let us observe the error probability. Define $\rho_{i}:=T_{K_{1}}^{*}(|i\rangle \langle i|)$. By direct calculations (see Appendix A for details), we have the following probability,

(37) $$\begin{array}{c} P_{\mathrm{err}(0;i,0;i)}=\frac{1}{2}\left(1-\langle i|\rho_{i}i\rangle \right). \end{array}$$

By using Equations (31) and (37), we have

(38) $$\begin{array}{ccc} F(\rho_{0},\rho_{1}) & = & \inf_{{\left(M_{a}\right)}_{a}:\mathrm{POVM}}\sum_{a}\sqrt{\mathrm{tr}\left(M_{a}\rho_{0}\right)\mathrm{tr}\left(M_{a}\rho_{1}\right)}\\ \; & \leq & \sqrt{\mathrm{tr}(|0\rangle \langle 0|\rho_{0})\mathrm{tr}(|0\rangle \langle 0|\rho_{1})}+\sqrt{\mathrm{tr}(|1\rangle \langle 1|\rho_{0})\mathrm{tr}(|1\rangle \langle 1|\rho_{1})}\\ \; & = & \sqrt{\langle 0|\rho_{0}0\rangle \left(1-\langle 1|\rho_{1}1\rangle \right)}+\sqrt{\left(1-\langle 0|\rho_{0}0\rangle \right)\langle 1|\rho_{1}1\rangle }\\ \; & \leq & \sqrt{1-\langle 1|\rho_{1}1\rangle }+\sqrt{1-\langle 0|\rho_{0}0\rangle }\\ \; & = & \sqrt{2P_{\mathrm{err}(0;0,0;0)}}+\sqrt{2P_{\mathrm{err}(0;1,0;1)}}, \end{array}$$

where we employ (|0〉〈0|,|1〉〈1|) as a POVM in the first inequality. Then, we have the trade-off inequality by the definition of trace distance, Equations (32) and (38). □

Let us consider that Eve tries to extract information from $H_{K_{1}}$ and $H_{K_{2}}$. Eve prepares a quantum systems $H_{E}$ in a quantum state Ω. She intercepts $H_{K_{1}}⊗H_{K_{2}}$ in the state $\rho^{\left(K_{1},K_{2}\right)}$ returned to Alice by King${}_{1}$ and King${}_{2}$. Then, she interacts both systems with $H_{E}$. Let us denote the interaction by

(39) $$K^{*}\left(\rho^{\left(K_{1},K_{2}\right)}\right):=V\rho^{\left(K_{1},K_{2}\right)}⊗\Omega V^{†},$$

where *V* is a unitary operator on $H_{K_{1}}⊗H_{K_{2}}⊗H_{E}$. And we denote the local state of $H_{E}$ (resp. $H_{K_{1}}⊗H_{K_{2}}$) by partial trace over the $H_{K_{1}}⊗H_{K_{2}}$ (resp. $H_{E}$)

(40) $$\begin{array}{c} K_{E}^{*}\left(\rho^{\left(K_{1},K_{2}\right)}\right):={\mathrm{tr}}_{H_{K_{1}}⊗H_{K_{2}}}K^{*}\left(\rho^{\left(K_{1},K_{2}\right)}\right)\;\;\left(\mathrm{resp}.\;K_{K_{1},K_{2}}^{*}\left(\rho^{\left(K_{1}K_{2}\right)}\right):={\mathrm{tr}}_{H_{K_{E}}}K^{*}\left(\rho^{\left(K_{1},K_{2}\right)}\right)\right). \end{array}$$

Let us consider that King${}_{1}$ and King${}_{2}$ perform the same measurement $M^{\left(1\right)}$ and obtain the same outcome *i*. Then, the state of $H_{K_{1}}⊗H_{K_{2}}$ before the interaction is $|\bar{i}\bar{i}\rangle \langle \bar{i}\bar{i}|$. Eve tries to extract information regarding to the secret key by distinguishing $K_{E}^{*}(|\bar{0}\bar{0}\rangle \langle \bar{0}\bar{0}|)$ and $K_{E}^{*}(|\bar{1}\bar{1}\rangle \langle \bar{1}\bar{1}|)$.

**Lemma** **2.** *The following relation between trace distance and fidelity holds,*

(41) $$\begin{array}{ccc} \left|\right|K_{E}^{*}(|\bar{0}\bar{0}\rangle \langle \bar{0}\bar{0}|)-K_{E}^{*}(|\bar{1}\bar{1}\rangle \langle \bar{1}\bar{1}|){\left|\right|}_{1}\; & \leq & \sum_{i\in \{0,1\}}F(K_{K_{1}K_{2}}^{*}(|ii\rangle \langle ii|),K_{K_{1}K_{2}}^{*}(|01\rangle \langle 01|)\\ \; & \; & +\sum_{i\in \{0,1\}}F(K_{K_{1}K_{2}}^{*}(|ii\rangle \langle ii|),K_{K_{1}K_{2}}^{*}(|10\rangle \langle 10|). \end{array}$$

**Proof of Lemma** **2.** From Lemma 3 in [27], we have

(42) $$|\langle i_{1}i_{2}\left|K\left(I⊗A\right)\right|i_{1}^{\prime }i_{2}^{\prime }\rangle |\leq ||A||F\left(K_{K_{1}K_{2}}^{*}(|i_{1}i_{2}\rangle \langle i_{1}i_{2}|),K_{K_{1}K_{2}}^{*}(|i_{1}^{\prime }i_{2}^{\prime }\rangle \langle i_{1}^{\prime }i_{2}^{\prime }|)\right)$$

for any operator *A* on $H_{E}$, where *K* is defined as $\mathrm{tr}K^{*}\left(\rho \right)X=\mathrm{tr}\rho K\left(X\right)$. By using Equation (42), we observe

(43) $$\begin{array}{ccc} |\mathrm{tr}[\left\{K_{E}^{*}(|\bar{0}\bar{0}\rangle \langle \bar{0}\bar{0}|)-K_{E}^{*}(|\bar{1}\bar{1}\rangle \langle \bar{1}\bar{1}|)\right\}A]|\; & = & |\mathrm{tr}\{(|\bar{0}\bar{0}\rangle \langle \bar{0}\bar{0}\left|-\right|\bar{1}\bar{1}\rangle \langle \bar{1}\bar{1}|)K\left(I⊗A\right)\}|\\ \; & = & |\mathrm{tr}\{\frac{1}{2}(|00\rangle \langle 01|+\left|00\rangle \langle 10\right|+\left|01\rangle \langle 00\right|+\left|01\rangle \langle 11\right|\\ \; & \; & +|10\rangle \langle 00|+|10\rangle \langle 11|+|11\rangle \langle 01|+|11\rangle \langle 10|)K(I⊗A)\}|\\ \; & \leq & \sum_{i\in \{0,1\}}\left|\langle ii|K\left(I⊗A\right)|01\rangle \right|+\sum_{i\in \{0,1\}}\left|\langle ii|K\left(I⊗A\right)|10\rangle \right|\\ \; & \leq & ||A||\{\sum_{i\in \{0,1\}}F(K_{K_{1}K_{2}}^{*}(|ii\rangle \langle ii|),K_{K_{1}K_{2}}^{*}(|01\rangle \langle 01|)\\ \; & \; & +\sum_{i\in \{0,1\}}F(K_{K_{1}K_{2}}^{*}(|ii\rangle \langle ii|),K_{K_{1}K_{2}}^{*}(|10\rangle \langle 10|)\}. \end{array}$$

In Equation (43), we take supreme over all *A* such that ||A||=1, then we have Equation (41). □

**Theorem** **2.** *The following trade-off inequality holds,*

(44) $$\begin{array}{ccc} D(K_{E}^{*}(|\bar{0}\bar{0}\rangle \langle \bar{0}\bar{0}|),K_{E}^{*}(|\bar{1}\bar{1}\rangle \langle \bar{1}\bar{1}|)) & < & \sum_{i_{1},i_{2}\in \left\{0,1\right\}}\sqrt{2P_{\mathrm{err}(0;i_{1},0;i_{2})}}. \end{array}$$

Although Eve tries to distinguish the states on $H_{K_{1}}⊗H_{K_{2}}$, this theorem gives the same claim as the one of Theorem 1. This theorem shows that Eve’s extracting information regarding kings’ keys related with the measurement $M^{\left(1\right)}$ inevitably induces disturbing the states and increases the error probability when both of kings choose the measurement $M^{\left(0\right)}$. Remark that similar inequalities between distinguishability of other pairs of states and the error probabilities can be proven in the similar way as below.

**Proof of Theorem** **2.** In the same manner, let us observe the error probability. Define $\rho_{i_{1}i_{2}}:=K_{K_{1}K_{2}}^{*}(|i_{1}i_{2}\rangle \langle i_{1}i_{2}|)$. By direct calculations (see Appendix B for details), we have the following probability,

(45) $$\begin{array}{c} P_{\mathrm{err}(0;i_{1},0;i_{2})}=\left\{\begin{array}{c} \frac{1}{2}(1-\langle i_{1}i_{2}|\rho_{i_{1}i_{2}}|i_{1}i_{2}\rangle )\;\left(i_{1}=i_{2}\right)\\ 1-\frac{1}{2}\langle i_{1}i_{2}|\rho_{i_{1}i_{2}}|i_{1}i_{2}\rangle \;\left(i_{1}\neq i_{2}\right). \end{array}\right. \end{array}$$

By using Equations (31) and (45), we have

(46) $$\begin{array}{ccc} F(\rho_{00},\rho_{01}) & = & \inf_{{\left(M_{a}\right)}_{a}:\mathrm{POVM}}\sum_{a}\sqrt{\mathrm{tr}\left(M_{a}\rho_{00}\right)\mathrm{tr}\left(M_{a}\rho_{01}\right)}\\ \; & \leq & \sqrt{\mathrm{tr}\{(|11\rangle \langle 11|+|01\rangle \langle 01|)\rho_{00}\}\mathrm{tr}\{(|00\rangle \langle 00|+|01\rangle \langle 01|)\rho_{01}\}}\\ \; & \; & +\sqrt{\mathrm{tr}\{(|00\rangle \langle 00|+|10\rangle \langle 10|)\rho_{00}\}\mathrm{tr}\{(|00\rangle \langle 00|+|10\rangle \langle 10|)\rho_{01}\}}\\ \; & < & \sqrt{\mathrm{tr}\{(|11\rangle \langle 11|+|01\rangle \langle 01|)\rho_{00}\}}+\sqrt{\mathrm{tr}\{(|00\rangle \langle 00|+|10\rangle \langle 10|)\rho_{01}\}}\\ \; & = & \sqrt{1-\langle 00|\rho_{00}|00\rangle -\langle 10|\rho_{00}|10\rangle }+\sqrt{1-\langle 01|\rho_{01}|01\rangle -\langle 11|\rho_{01}|11\rangle }\\ \; & < & \sqrt{1-\langle 00|\rho_{00}|00\rangle }+\sqrt{2-\langle 01|\rho_{01}|01\rangle }\\ \; & = & \sqrt{2P_{\mathrm{err}(0;0,0;0)}}+\sqrt{2P_{\mathrm{err}(0;0,0;1)}}. \end{array}$$

where we employ (|11〉〈11|+|01〉〈01|,|00〉〈00|+|10〉〈10|) as a POVM in the first inequality. In the same manner, we have

(47) $$\begin{array}{ccc} F(\rho_{ii},\rho_{01}) & < & \sqrt{2P_{\mathrm{err}(0;i,0;i)}}+\sqrt{2P_{\mathrm{err}(0;0,0;1)}}, \end{array}$$

(48) $$\begin{array}{ccc} F(\rho_{ii},\rho_{10}) & < & \sqrt{2P_{\mathrm{err}(0;i,0;i)}}+\sqrt{2P_{\mathrm{err}(0;1,0;0)}}\;\left(i\in \left\{0,1\right\}\right). \end{array}$$

Then, we have the trade-off inequality by the definition of trace distance, Equations (41) and (48). □

## 5. Summary

In this paper, we discussed the quantum key distribution protocol using the mean multi-kings’ problem. By using the protocol, Alice can share the secret key with King${}_{j}$ (j=1,2,…,n). In the case of n=2, we considered whether Eve can extract information when she can performs the interaction between her own quantum system and the qubit returned by King${}_{j}$ and can performs any measurement on her quantum system at any time. We employed trace distance as a measure for distinguishability of the states for Eve. Furthermore, we gave the trade-off inequalities between trace distance of the quantum states corresponding to the secret key for Eve and the error probability which represents probability that the bit sequences shared by the legitimate users do not match. In BB84, such relation is know as the information disturbance theorem and the theorem is also regarded as an information theoretical version of the uncertainty relation. Our inequalities showed that Eve’s extracting information regarding kings’ keys inevitably induces disturbing the states and increases the error probability even though Alice can use the post-information to guess kings’ outcomes. This implies that the information gain by Eve increases possibility for the legitimate users to detect the existence of the attacks. In particular, when the corresponding error probability is zero, Eve cannot extract any information.

## Figures and Tables

**Table 1:** The relationship among kings’ measurements $J_{1},J_{2}$, Alice’s outcome *k*, and kings’ outcomes $i_{1},i_{2}$ when she chooses $|\Phi^{\left(W\right)}\rangle$. In this table, NA means that probability of obtaining the corresponding outcome *k* is zero unless Eve performs an attack because the corresponding matrix $P_{k}^{\left(W\right)}$ is a zero matrix. An example of Alice’s guessing: Alice is assured that kings’ outcome $\left(i_{1},i_{2}\right)$ is (0,0) when $W=Z,J_{1}=0,J_{2}=0$, and k=(0,0,0,0).

$W=Z,J_{1}=0,J_{2}=0$		$W=Z,J_{1}=0,J_{2}=1$		$W=Z,J_{1}=1,J_{2}=0$		$W=Z,J_{1}=1,J_{2}=1$	
$W=X,J_{1}=1,J_{2}=1$		$W=X,J_{1}=1,J_{2}=0$		$W=X,J_{1}=0,J_{2}=1$		$W=X,J_{1}=0,J_{2}=0$	
k	$\left(i_{1},i_{2}\right)$	k	$\left(i_{1},i_{2}\right)$	k	$\left(i_{1},i_{2}\right)$	k	$\left(i_{1},i_{2}\right)$
(0,0,0,0)	(0,0)	(0,0,0,0)	(0,0)	(0,0,0,0)	(0,0)	(0,0,0,0)	(0,0)
(0,0,0,1) NA	——	(0,0,0,1) NA	——	(0,0,0,1) NA	——	(0,0,0,1) NA	——
(0,0,1,0)	(0,0)	(0,0,1,0)	(0,1)	(0,0,1,0)	(0,0)	(0,0,1,0)	(0,1)
(0,0,1,1) NA	——	(0,0,1,1) NA	——	(0,0,1,1) NA	——	(0,0,1,1) NA	——
(0,1,0,0) NA	——	(0,1,0,0) NA	——	(0,1,0,0) NA	——	(0,1,0,0) NA	——
(0,1,0,1)	(1,1)	(0,1,0,1)	(1,0)	(0,1,0,1)	(0,1)	(0,1,0,1)	(0,0)
(0,1,1,0) NA	——	(0,1,1,0) NA	——	(0,1,1,0) NA	——	(0,1,1,0) NA	——
(0,1,1,1)	(1,1)	(0,1,1,1)	(1,1)	(0,1,1,1)	(0,1)	(0,1,1,1)	(0,1)
(1,0,0,0)	(0,0)	(1,0,0,0)	(0,0)	(1,0,0,0)	(1,0)	(1,0,0,0)	(1,0)
(1,0,0,1) NA	——	(1,0,0,1) NA	——	(1,0,0,1) NA	——	(1,0,0,1) NA	——
(1,0,1,0)	(0,0)	(1,0,1,0)	(0,1)	(1,0,1,0)	(1,0)	(1,0,1,0)	(1,1)
(1,0,1,1) NA	——	(1,0,1,1) NA	——	(1,0,1,1) NA	——	(1,0,1,1) NA	——
(1,1,0,0) NA	——	(1,1,0,0) NA	——	(1,1,0,0) NA	——	(1,1,0,0) NA	——
(1,1,0,1)	(1,1)	(1,1,0,1)	(1,0)	(1,1,0,1)	(1,1)	(1,1,0,1)	(1,0)
(1,1,1,0) NA	——	(1,1,1,0) NA	——	(1,1,1,0) NA	——	(1,1,1,0) NA	——
(1,1,1,1)	(1,1)	(1,1,1,1)	(1,1)	(1,1,1,1)	(1,1)	(1,1,1,1)	(1,1)

## Appendix A

We provide a direct calculation for obtaining the error probabilities in the proof of Theorem 1. Let us consider that the initial state is $|\Phi^{\left(W\right)}\rangle$, King${}_{j}$ (j∈{1,2}) obtains an outcome $i_{j}$ with $M^{\left(J_{j}\right)}$, and Eve performs the interaction on $H_{K_{1}}⊗H_{E}$. Let $\rho_{\left(J_{1};i_{1},J_{2};i_{2}\right)}^{\left(W\right)}$ be a state of the composite system before Alice’s measurement. The state takes one of the following forms,

(A1) $$\begin{array}{ccc} \rho_{\left(0;i_{1},0;i_{1}\right)}^{\left(Z\right)} & = & |i_{1}\rangle \langle i_{1}|⊗\rho_{i_{1}}⊗|i_{1}\rangle \langle i_{1}|, \end{array}$$

(A2) $$\begin{array}{ccc} \rho_{\left(0;i_{1},1;i_{2}\right)}^{\left(Z\right)} & = & |i_{1}\rangle \langle i_{1}|⊗\rho_{i_{1}}⊗|{\bar{i}}_{2}\rangle \langle {\bar{i}}_{2}|, \end{array}$$

(A3) $$\begin{array}{ccc} \rho_{\left(1;i_{1},0;i_{2}\right)}^{\left(Z\right)} & = & |i_{2}\rangle \langle i_{2}|⊗\rho_{{\bar{i}}_{1}}⊗|i_{2}\rangle \langle i_{2}|, \end{array}$$

(A4) $$\begin{array}{ccc} \rho_{\left(1;i_{1},1;i_{2}\right)}^{\left(Z\right)} & = & |{\bar{i}}_{1}\rangle \langle {\bar{i}}_{1}|⊗\rho_{{\bar{i}}_{1}}⊗|{\bar{i}}_{2}\rangle \langle {\bar{i}}_{2}|, \end{array}$$

(A5) $$\begin{array}{ccc} \rho_{\left(0;i_{1},0;i_{2}\right)}^{\left(X\right)} & = & |i_{1}⊕i_{2}\rangle \langle i_{1}⊕i_{2}|⊗\rho_{i_{1}}⊗|i_{2}\rangle \langle i_{2}|, \end{array}$$

(A6) $$\begin{array}{ccc} \rho_{\left(0;i_{1},1;i_{2}\right)}^{\left(X\right)} & = & |{\bar{i}}_{2}\rangle \langle {\bar{i}}_{2}|⊗\rho_{i_{1}}⊗|{\bar{i}}_{2}\rangle \langle {\bar{i}}_{2}|, \end{array}$$

(A7) $$\begin{array}{ccc} \rho_{\left(1;i_{1},0;i_{2}\right)}^{\left(X\right)} & = & |{\bar{i}}_{1}\rangle \langle {\bar{i}}_{1}|⊗\rho_{{\bar{i}}_{1}}⊗|i_{2}\rangle \langle i_{2}|, \end{array}$$

(A8) $$\begin{array}{ccc} \rho_{\left(1;i_{1},1;i_{1}\right)}^{\left(X\right)} & = & |{\bar{i}}_{1}\rangle \langle {\bar{i}}_{1}|⊗\rho_{{\bar{i}}_{1}}⊗|{\bar{i}}_{1}\rangle \langle {\bar{i}}_{1}|, \end{array}$$

where $\rho_{i}:=T_{K_{1}}^{*}(|i\rangle \langle i|)$, $\rho_{\bar{i}}:=T_{K_{1}}^{*}(|\bar{i}\rangle \langle \bar{i}|)$, and ⊕ denotes exclusive or.

By direct calculation of

(A9) $$P_{\mathrm{suc}(J_{1};i_{1},J_{2};i_{2})}^{\left(W\right)}=\sum_{k\in S_{{\left(J_{j},i_{j}\right)}_{j=1}^{2}}^{\left(W\right)}}\mathrm{tr}\left(P_{k}^{\left(W\right)}\rho_{\left(J_{1};i_{1},J_{2};i_{2}\right)}^{\left(W\right)}\right),$$

we have the following probabilities,

(A10) $$\begin{array}{ccc} & & P_{\mathrm{suc}(0;i_{1},0;i_{1})}^{\left(Z\right)}=P_{\mathrm{suc}(0;i_{1},1;i_{2})}^{\left(Z\right)}=1, \end{array}$$

(A11) $$\begin{array}{ccc} & & P_{\mathrm{suc}(1;i_{1},0;i_{2})}^{\left(Z\right)}=P_{\mathrm{suc}(1;i_{1},1;i_{2})}^{\left(Z\right)}=\langle {\bar{i}}_{1}|\rho_{{\bar{i}}_{1}}{\bar{i}}_{1}\rangle , \end{array}$$

(A12) $$\begin{array}{ccc} & & P_{\mathrm{suc}(0;i_{1},0;i_{2})}^{\left(X\right)}=P_{\mathrm{suc}(0;i_{1},1;i_{2})}^{\left(X\right)}=\langle i_{1}|\rho_{i_{1}}i_{1}\rangle , \end{array}$$

(A13) $$\begin{array}{ccc} & & P_{\mathrm{suc}(1;i_{1},0;i_{2})}^{\left(X\right)}=P_{\mathrm{suc}(1;i_{1},1;i_{1})}^{\left(X\right)}=1, \end{array}$$

where we can find out the index set $S_{{\left(J_{j},i_{j}\right)}_{j=1}^{2}}^{\left(W\right)}$ in Table 1. By the definition of $P_{\mathrm{suc}(J_{1};i_{1},J_{2};i_{2})}$, we have the following probabilities,

(A14) $$\begin{array}{ccc} P_{\mathrm{suc}(0;i_{1},0;i_{2})} & = & \left\{\begin{array}{c} \frac{1}{2}\left(\langle i_{1}|\rho_{i_{1}}i_{1}\rangle +1\right)\;\left(i_{1}=i_{2}\right)\\ \frac{1}{2}\langle i_{1}|\rho_{i_{1}}i_{1}\rangle \;\left(i_{1}\neq i_{2}\right), \end{array}\right. \end{array}$$

(A15) $$\begin{array}{ccc} P_{\mathrm{suc}(0;i_{1},1;i_{2})} & = & \frac{1}{2}\langle i_{1}|\rho_{i_{1}}i_{1}\rangle , \end{array}$$

(A16) $$\begin{array}{ccc} P_{\mathrm{suc}(1;i_{1},0;i_{2})} & = & \frac{1}{2}\langle {\bar{i}}_{1}|\rho_{{\bar{i}}_{1}}{\bar{i}}_{1}\rangle , \end{array}$$

(A17) $$\begin{array}{ccc} P_{\mathrm{suc}(1;i_{1},1;i_{2})} & = & \left\{\begin{array}{c} \frac{1}{2}\left(\langle {\bar{i}}_{1}|\rho_{{\bar{i}}_{1}}{\bar{i}}_{1}\rangle +1\right)\;\left(i_{1}=i_{2}\right)\\ \frac{1}{2}\langle {\bar{i}}_{1}|\rho_{{\bar{i}}_{1}}{\bar{i}}_{1}\rangle \;\left(i_{1}\neq i_{2}\right). \end{array}\right. \end{array}$$

Then, we can observe the error probabilities from these probabilities.

## Appendix B

We provide a direct calculation for obtaining the error probabilities in the proof of Theorem 2. Let us consider that the initial state is $|\Phi^{\left(W\right)}\rangle$, King${}_{j}$ (j∈{1,2}) obtains an outcome $i_{j}$ with $M^{\left(J_{j}\right)}$, and Eve performs the interaction on $H_{K_{1}}⊗H_{K_{2}}⊗H_{E_{j}}$. Let ${\rho \prime }_{\left(J_{1};i_{1},J_{2};i_{2}\right)}^{\left(W\right)}$ be a state of the composite system before Alice’s measurement. The state takes one of the following forms,

(A18) $$\begin{array}{ccc} {\rho \prime }_{\left(0;i_{1},0;i_{1}\right)}^{\left(Z\right)} & = & |i_{1}\rangle \langle i_{1}|⊗\rho_{i_{1}i_{1}}, \end{array}$$

(A19) $$\begin{array}{ccc} {\rho \prime }_{\left(0;i_{1},1;i_{2}\right)}^{\left(Z\right)} & = & |i_{1}\rangle \langle i_{1}|⊗\rho_{i_{1}{\bar{i}}_{2}}, \end{array}$$

(A20) $$\begin{array}{ccc} {\rho \prime }_{\left(1;i_{1},0;i_{2}\right)}^{\left(Z\right)} & = & |i_{2}\rangle \langle i_{2}|⊗\rho_{{\bar{i}}_{1}i_{2}}, \end{array}$$

(A21) $$\begin{array}{ccc} {\rho \prime }_{\left(1;i_{1},1;i_{2}\right)}^{\left(Z\right)} & = & |{\bar{i}}_{1}\rangle \langle {\bar{i}}_{1}|⊗\rho_{{\bar{i}}_{1}{\bar{i}}_{2}}, \end{array}$$

(A22) $$\begin{array}{ccc} {\rho \prime }_{\left(0;i_{1},0;i_{2}\right)}^{\left(X\right)} & = & |i_{1}⊕i_{2}\rangle \langle i_{1}⊕i_{2}|⊗\rho_{i_{1}i_{2}}, \end{array}$$

(A23) $$\begin{array}{ccc} {\rho \prime }_{\left(0;i_{1},1;i_{2}\right)}^{\left(X\right)} & = & |{\bar{i}}_{2}\rangle \langle {\bar{i}}_{2}|⊗\rho_{{\bar{i}}_{1}i_{2}}, \end{array}$$

(A24) $$\begin{array}{ccc} {\rho \prime }_{\left(1;i_{1},0;i_{2}\right)}^{\left(X\right)} & = & |{\bar{i}}_{1}\rangle \langle {\bar{i}}_{1}|⊗\rho_{{\bar{i}}_{1}i_{2}}, \end{array}$$

(A25) $$\begin{array}{ccc} {\rho \prime }_{\left(1;i_{1},1;i_{1}\right)}^{\left(X\right)} & = & |{\bar{i}}_{1}\rangle \langle {\bar{i}}_{1}|⊗\rho_{{\bar{i}}_{1}{\bar{i}}_{1}}, \end{array}$$

where $\rho_{ij}:=K_{K_{1}K_{2}}^{*}(|ij\rangle \langle ij|)$ ($i,j\in \{0,1,\bar{0},\bar{1}\}$).

By direct calculation of

(A26) $$P_{\mathrm{suc}(J_{1};i_{1},J_{2};i_{2})}^{\left(W\right)}=\sum_{k\in S_{{\left(J_{j},i_{j}\right)}_{j=1}^{2}}^{\left(W\right)}}\mathrm{tr}\left(P_{k}^{\left(W\right)}{\rho \prime }_{\left(J_{1};i_{1},J_{2};i_{2}\right)}^{\left(W\right)}\right),$$

we have the following probabilities,

(A27) $$\begin{array}{ccc} P_{\mathrm{suc}(0;i_{1},0;i_{1})}^{\left(Z\right)} & = & 1, \end{array}$$

(A28) $$\begin{array}{ccc} P_{\mathrm{suc}(0;i_{1},1;i_{2})}^{\left(Z\right)} & = & \langle {\bar{i}}_{1}{\bar{i}}_{2}|\rho_{i_{1}{\bar{i}}_{2}}|{\bar{i}}_{1}{\bar{i}}_{2}\rangle +\langle \bar{i_{1}⊕1}{\bar{i}}_{2}|\rho_{i_{1}{\bar{i}}_{2}}|\bar{i_{1}⊕1}{\bar{i}}_{2}\rangle , \end{array}$$

(A29) $$\begin{array}{ccc} P_{\mathrm{suc}(1;i_{1},0;i_{2})}^{\left(Z\right)} & = & \langle {\bar{i}}_{1}{\bar{i}}_{2}|\rho_{{\bar{i}}_{1}i_{2}}|{\bar{i}}_{1}{\bar{i}}_{2}\rangle +\langle {\bar{i}}_{1}\bar{i_{2}⊕1}|\rho_{{\bar{i}}_{1}i_{2}}|{\bar{i}}_{1}\bar{i_{2}⊕1}\rangle , \end{array}$$

(A30) $$\begin{array}{ccc} P_{\mathrm{suc}(1;i_{1},1;i_{2})}^{\left(Z\right)} & = & \langle {\bar{i}}_{1}{\bar{i}}_{2}|\rho_{{\bar{i}}_{1}{\bar{i}}_{2}}|{\bar{i}}_{1}{\bar{i}}_{2}\rangle , \end{array}$$

(A31) $$\begin{array}{ccc} P_{\mathrm{suc}(0;i_{1},0;i_{2})}^{\left(X\right)} & = & \langle i_{1}i_{2}|\rho_{i_{1}i_{2}}|i_{1}i_{2}\rangle , \end{array}$$

(A32) $$\begin{array}{ccc} P_{\mathrm{suc}(0;i_{1},1;i_{2})}^{\left(X\right)} & = & \langle i_{1}i_{2}|\rho_{i_{1}{\bar{i}}_{2}}|i_{1}i_{2}\rangle +\langle i_{1}i_{2}⊕1|\rho_{i_{1}{\bar{i}}_{2}}|i_{1}i_{2}⊕1\rangle , \end{array}$$

(A33) $$\begin{array}{ccc} P_{\mathrm{suc}(1;i_{1},0;i_{2})}^{\left(X\right)} & = & \langle i_{1}i_{2}|\rho_{{\bar{i}}_{1}i_{2}}|i_{1}i_{2}\rangle +\langle i_{1}⊕1i_{2}|\rho_{{\bar{i}}_{1}i_{2}}|i_{1}⊕1i_{2}\rangle , \end{array}$$

(A34) $$\begin{array}{ccc} P_{\mathrm{suc}(1;i_{1},1;i_{1})}^{\left(X\right)} & = & 1. \end{array}$$

By the definition of $P_{\mathrm{suc}(J_{1};i_{1},J_{2};i_{2})}$, we have the following probabilities,

(A35) $$\begin{array}{ccc} P_{\mathrm{suc}(0;i_{1},0;i_{2})} & = & \left\{\begin{array}{c} \frac{1}{2}(\langle i_{1}i_{2}|\rho_{i_{1}i_{2}}|i_{1}i_{2}\rangle +1)\;\left(i_{1}=i_{2}\right)\\ \frac{1}{2}\langle i_{1}i_{2}|\rho_{i_{1}i_{2}}|i_{1}i_{2}\rangle \;\left(i_{1}\neq i_{2}\right), \end{array}\right. \end{array}$$

(A36) $$\begin{array}{ccc} P_{\mathrm{suc}(0;i_{1},1;i_{2})} & = & \frac{1}{2}(\langle {\bar{i}}_{1}{\bar{i}}_{2}|\rho_{i_{1}{\bar{i}}_{2}}|{\bar{i}}_{1}{\bar{i}}_{2}\rangle +\langle \bar{i_{1}⊕1}{\bar{i}}_{2}|\rho_{i_{1}{\bar{i}}_{2}}|\bar{i_{1}⊕1}{\bar{i}}_{2}\rangle \\ & & +\langle i_{1}i_{2}|\rho_{i_{1}{\bar{i}}_{2}}|i_{1}i_{2}\rangle +\langle i_{1}i_{2}⊕1|\rho_{i_{1}{\bar{i}}_{2}}|i_{1}i_{2}⊕1\rangle ), \end{array}$$

(A37) $$\begin{array}{ccc} P_{\mathrm{suc}(1;i_{1},0;i_{2})} & = & \frac{1}{2}(\langle {\bar{i}}_{1}{\bar{i}}_{2}|\rho_{{\bar{i}}_{1}i_{2}}|{\bar{i}}_{1}{\bar{i}}_{2}\rangle +\langle {\bar{i}}_{1}\bar{i_{2}⊕1}|\rho_{{\bar{i}}_{1}i_{2}}|{\bar{i}}_{1}\bar{i_{2}⊕1}\rangle \\ & & +\langle i_{1}i_{2}|\rho_{{\bar{i}}_{1}i_{2}}|i_{1}i_{2}\rangle +\langle i_{1}⊕1i_{2}|\rho_{{\bar{i}}_{1}i_{2}}|i_{1}⊕1i_{2}\rangle ), \end{array}$$

(A38) $$\begin{array}{ccc} P_{\mathrm{suc}(1;i_{1},1;i_{2})} & = & \left\{\begin{array}{c} \frac{1}{2}(\langle {\bar{i}}_{1}{\bar{i}}_{2}|\rho_{{\bar{i}}_{1}{\bar{i}}_{2}}|{\bar{i}}_{1}{\bar{i}}_{2}\rangle +1)\;\left(i_{1}=i_{2}\right)\\ \frac{1}{2}\langle {\bar{i}}_{1}{\bar{i}}_{2}|\rho_{{\bar{i}}_{1}{\bar{i}}_{2}}|{\bar{i}}_{1}{\bar{i}}_{2}\rangle \;\left(i_{1}\neq i_{2}\right). \end{array}\right. \end{array}$$

Then, we can observe the error probabilities from those probabilities.
